# Chikungunya virus entry is strongly inhibited by phospholipase A2 isolated from the venom of *Crotalus durissus terrificus*

**DOI:** 10.1038/s41598-021-88039-4

**Published:** 2021-04-22

**Authors:** Igor Andrade Santos, Jacqueline Farinha Shimizu, Débora Moraes de Oliveira, Daniel Oliveira Silva Martins, Léia Cardoso-Sousa, Adélia Cristina Oliveira Cintra, Victor Hugo Aquino, Suely Vilela Sampaio, Nilson Nicolau-Junior, Robinson Sabino-Silva, Andres Merits, Mark Harris, Ana Carolina Gomes Jardim

**Affiliations:** 1 Institute of Biomedical Science (ICBIM), Federal University of Uberlândia (UFU), Avenida Amazonas, 4C- Room 216, Umuarama, Uberlândia, Minas Gerais CEP: 38405-302 Brazil; 2 Institute of Biosciences, Humanities and Exact Sciences (Ibilce), São Paulo State University (Unesp), Campus São José do Rio Preto, São José do Rio Preto, SP Brazil; 3 Department of Clinical, Toxicological and Bromatological Analyses, School of Pharmaceutical Sciences of Ribeirao Preto, University of São Paulo (USP), Ribeirão Preto, SP Brazil; 4 Institute of Biotechnology, Federal University of Uberlândia (UFU), Uberlândia, MG Brazil; 5 Institute of Technology, University of Tartu, Tartu, Estonia; 6 Faculty of Biological Sciences and Astbury Centre for Structural Molecular Biology, University of Leeds, Leeds, UK

**Keywords:** Microbiology, Virology, Alphaviruses, Antivirals

## Abstract

Chikungunya virus (CHIKV) is the etiologic agent of Chikungunya fever, a globally spreading mosquito-borne disease. There is no approved antiviral or vaccine against CHIKV, highlighting an urgent need for novel therapies. In this context, snake venom proteins have demonstrated antiviral activity against several viruses, including arboviruses which are relevant to public health. In particular, the phospholipase A2_CB_ (PLA2_CB_), a protein isolated from the venom of *Crotalus durissus terrificus* was previously shown to possess anti-inflammatory, antiparasitic, antibacterial and antiviral activities. In this study, we investigated the multiple effects of PLA2_CB_ on the CHIKV replicative cycle in BHK-21 cells using CHIKV-*nanoluc*, a marker virus carrying *nanoluciferase* reporter. The results demonstrated that PLA2_CB_ possess a strong anti-CHIKV activity with a selectivity index of 128. We identified that PLA2_CB_ treatment protected cells against CHIKV infection, strongly impairing virus entry by reducing adsorption and post-attachment stages. Moreover, PLA2_CB_ presented a modest yet significant activity towards post-entry stages of CHIKV replicative cycle. Molecular docking calculations indicated that PLA2_CB_ may interact with CHIKV glycoproteins, mainly with E1 through hydrophobic interactions. In addition, infrared spectroscopy measurements indicated interactions of PLA2_CB_ and CHIKV glycoproteins, corroborating with data from in silico analyses. Collectively, this data demonstrated the multiple antiviral effects of PLA2_CB_ on the CHIKV replicative cycle, and suggest that PLA2_CB_ interacts with CHIKV glycoproteins and that this interaction blocks binding of CHIKV virions to the host cells.

## Introduction

Chikungunya virus (CHIKV), a member of the genus *Alphavirus*, family *Togaviridae*^1^, is the causative agent of Chikungunya fever^2^. CHIKV virions comprise an icosahedral capsid with a positive single stranded RNA genome of approximately 12 kb^3^ surrounded by a lipid envelope with E1, E2 and E3 glycoproteins in its surface^4,5^.

CHIKV was first identified in 1950 in Tanzania, Africa and related to Chikungunya fever in 1955^6,7^. CHIKV is transmitted through the bite of *Aedes aegypti* and *Aedes albopictus* mosquito^8,9^, and, therefore, have been associated to the epidemics in tropical and subtropical regions^10^. Since then, CHIKV outbreaks have been identified in many regions including islands of Indian Ocean, India, South-East Asia, France, Italy and the Americas^11^. In Brazil, the first cases of Chikungunya fever were documented in 2014, and since then, the disease became an endemic^12^. From January to June of 2020, 48,316 cases and 11 deaths by CHIKV were notified^13^.

Chikungunya fever symptoms include fever, nausea, fatigue, arthralgia and polyarthralgia^14^. In some rare cases, infected individuals can develop hepatitis, myocarditis, and encephalopathy, ultimately leading to death of patients^14,15^. Unlike other arboviruses, CHIKV infection can result in chronic symptoms lasting for months or years, resulting in a disabling disease^16,17^. There are no approved antiviral drugs against CHIKV infection, as a consequence the treatment is often palliative and symptomatic, based on analgesics, non-steroidal anti-inflammatory, rest, and hydration^18^.

Given that many approved drugs employed in the treatment of infectious and chronic diseases originated or derived from natural sources^19,20^, it is reasonable to hypothesize that natural compounds may also be exploited to generate antiviral drugs. In this context, proteins isolated from snake venoms represent promising drug leads, since they are a complex mixture of lectins, oxidases, disintegrins, metalloproteins, and phospholipases A2 (PLA2s)^21,22^. From these, PLA2s, in its turn, are members of a secreted phospholipases, which can act in the cell membranes and play several roles in biological systems^23–25^.

The snake venom isolated from *Crotalus durissus terrificus* has numerous constituents such as crotoxin, crotamin, neurotoxin, among others^26,27^. Crotoxin is the major constituent of the *C. d. terrificus* venom. It is characterized as a protein complex composed by two noncovalent subunits, the basic subunit phospholipase A2 (PLA2_CB_), and the acid subunit crotapotin^28,29^. Subsequently, PLA2_CB_ is approximately 14 kDa protein which possess anti-inflammatory, antiparasitic, and antibacterial properties^30,31^. PLA2_CB_ has also presented activity towards viruses such as hepacivirus C (HCV)^32^, Rocio (ROCV), Mayaro (MAYV), Dengue (DENV) and Yellow Fever (YFV)^33,34^. Russo and coworkers expressed and purified two recombinant PLA2_CB_ (rPLA2_CB_) and partially assessed its anti-CHIKV activity. It was found that rPLA2_CB_ proteins possess lower antiviral activity and higher cytotoxicity profile than the native protein, probably due to nine additional amino acid residues present in their sequences^35^. Considering these previous results, herein we performed thorough in vitro evaluation of the effects of the native PLA2_CB_ on the CHIKV replication cycle.

## Results

### PLA2_CB_ strongly impairs CHIKV infection in vitro

**Figure 1:**
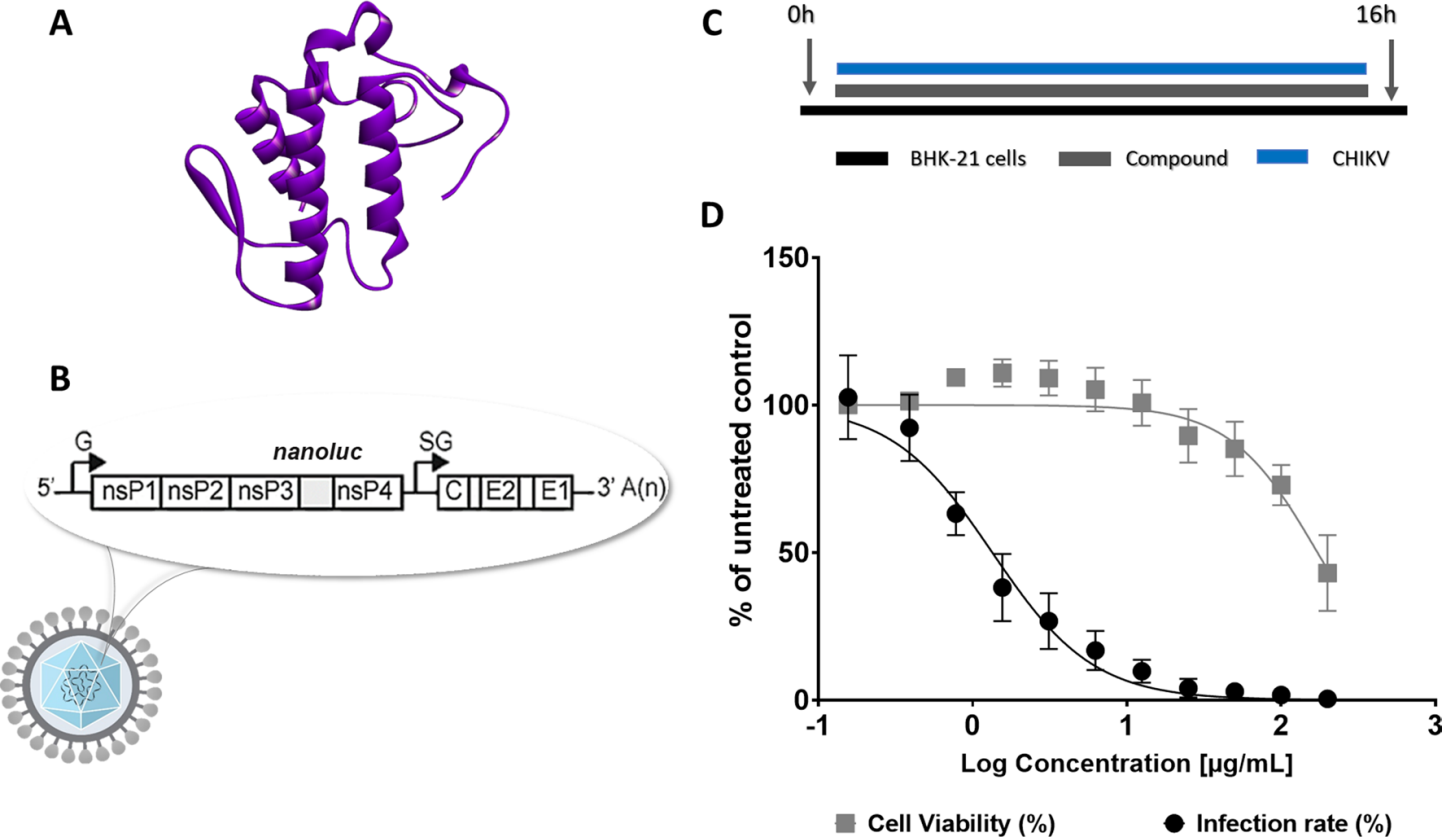
The activity of Phospholipase A2_CB_ (PLA2_CB_) against CHIKV infection. (**A**) Phospholipase A2_CB_ protein structure (PDB: 3R0L). (**B**) Schematic representation of CHIKV*-nanoluc* genome. (**C**) Schematic description of infectivity assay. (**D**) BHK-21 cells were treated with concentrations of PLA2_CB_ ranging from 0.195 to 200 µg/mL. CHIKV replication was quantified by measuring of *nanoluciferase* activity (indicated by black dot) and cellular viability was measured using an MTT assay (indicated by grey square). The mean values of three independent experiments each measured in triplicate including the standard deviation are shown. All images were generated using GraphPad Prism 8 and GIMP 2.10.20 (www.gimp.org).

We investigated the anti-CHIKV activity of the PLA2_CB_ (Fig. 1A) using BHK-21 cells and a recombinant CHIKV that expresses a *nanoluciferase* reporter (CHIKV*-nanoluc*) (Fig. 1B)^36,37^. First, the PLA2_CB_ antiviral activity was evaluated by performing a dose–response assay to determine the effective concentration of 50% (EC_50_) and cytotoxicity of 50% (CC_50_). BHK-21 cells were infected with CHIKV-*nanoluc* and simultaneously treated with PLA2_CB_ at concentrations ranging from 0.195 to 200 µg/mL in two-fold serial dilutions, and viral replication was assessed 16 h post-infection (h.p.i.) (Fig. 1C). In parallel, cell viability was assessed by an MTT assay. PLA2_CB_ was found to be able to inhibit virus replication to greater than 99%, while the cell viability at the highest concentration tested was 43%. It was determined that PLA2_CB_ has the EC50 of 1.34 µg/mL, CC50 of 172 µg/mL, and the Selectivity Index (SI) of 128 (Fig. 1D). Thus, PLA2_CB_ acts as strongly inhibitor of CHIKV infection with high SI value.

### PLA2_CB_ strongly inhibits early stages of CHIKV infection

Time-of-addition type of experiments were used to analyze the effect of PLA2_CB_ on different stages of CHIKV replication. For all of these assays, cells were treated with PLA2_CB_ at 12.5 µg/mL, a concentration that inhibited virus replication by ~ 91% without affecting cell viability (Fig. 1D).

**Figure 2:**
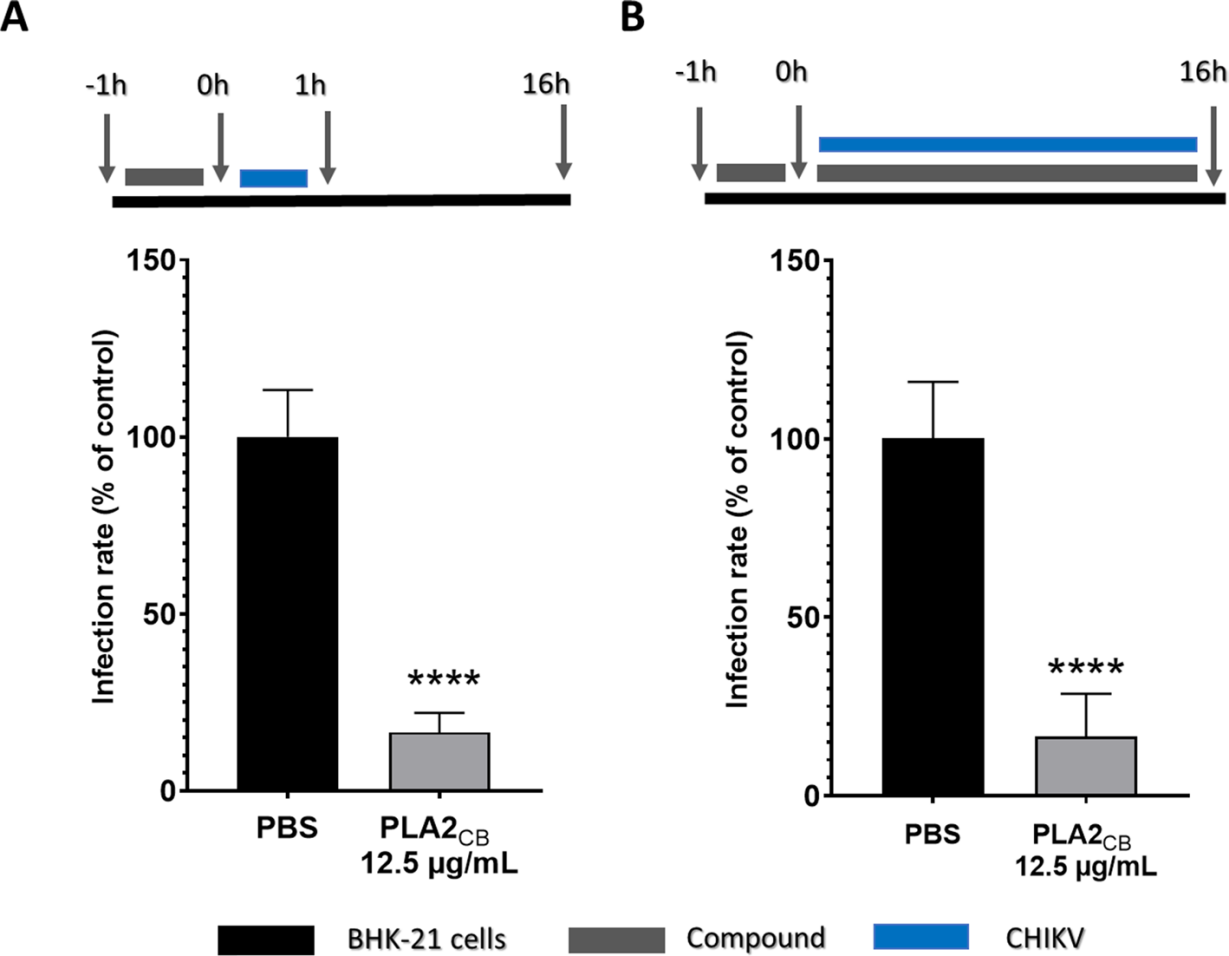
PLA2_CB_ protects cells against CHIKV infection. (**A**) BHK-21 cells were treated with PLA2_CB_ at 12.5 μg/mL for 1 h. Then, cells were washed and infected with CHIKV-*nanoluc* at MOI 0.1 for 1 h. The compound-containing medium was removed and replaced with a fresh medium. (**B**) BHK-21 cells were treated with the PLA2_CB_ at 12.5 μg/mL for 1 h, then were infected with CHIKV-*nanoluc* in the presence of PLA2_CB_. For both assays, CHIKV replication was measured by *nanoluc* activity at 16 h.p.i.. Schematic representation of each time-based assay as indicated by BHK-21 cells (black bars), PLA2_CB_ (grey bars), and CHIKV-*nanoluc* (blue bars). Mean values ± SD of a minimum of three independent experiments each measured in triplicate. (****) P < 0.0001. All images were generated using GraphPad Prism 8 and GIMP 2.10.20 (www.gimp.org).

To assess the protective effects of PLA2_CB_ against CHIKV infection, cells were pretreated with PLA2_CB_ for 1 h at 37 °C, washed extensively with PBS to remove the compound and infected with CHIKV-*nanoluc* for 1 h. Then, the supernatant was removed, cells were added of fresh medium and luciferase levels were measured 16 h.p.i. (Fig. 2A). PLA2_CB_ significantly reduced CHIKV-*nanoluc* infection by 84% (p < 0.01), demonstrating a robust protective effect (Fig. 2A). The protective effect did not increase when the compounds was present for all duration of the experiment (Fig. 2B), demonstrating that pre-treatment of cells with PLA2_CB_ inhibited CHIKV replication. This data suggests that PLA2_CB_ acts by protecting cells against infection and/or by affecting early stages of CHIKV infection.

**Figure 3:**
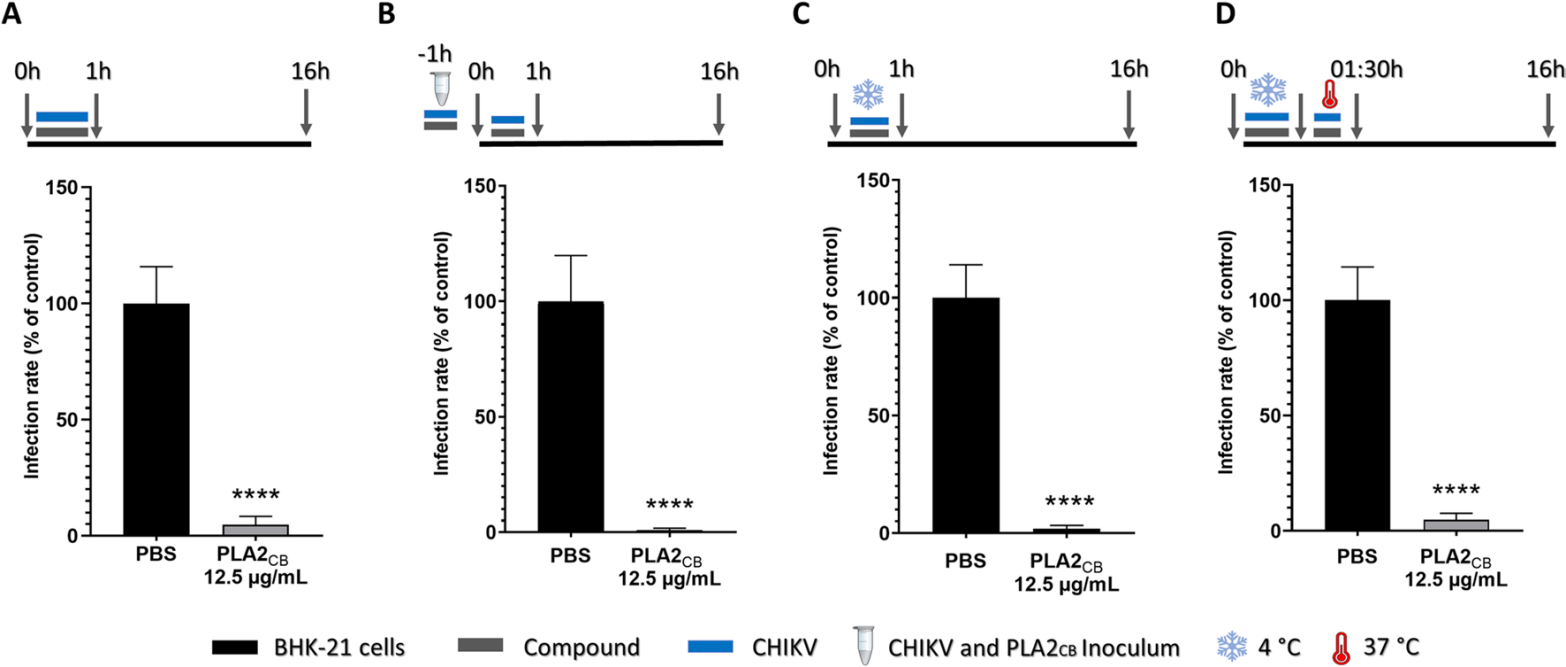
PLA2_CB_ has virucidal activity and blocks CHIKV entry to the host cells. (**A**) BHK-21 cells were infected with CHIKV-*nanoluc* (MOI 0.1) and simultaneously treated with PLA2_CB_ at 12.5 μg/mL for 1 h. Cells were washed and replaced by fresh medium. (**B**) CHIKV-*nanoluc* and PLA2_CB_ at 12.5 μg/mL were incubated for 1 h at 37 °C and then for one extra hour with the cells. Then, virus and compound were removed, cells were washed with PBS, and added fresh medium. (**C**) BHK-21 cells were infected with the virus and simultaneously treated with PLA2_CB_ at 12.5 μg/mL for 1 h at 4 °C. The cells were washed to remove virus and compound and replaced with fresh medium. (**D**) BHK-21 cells were infected with virus and simultaneously treated with PLA2_CB_ at 12.5 μg/mL for 1 h at 4 °C. Then, cells were incubated for additional 30 min with compound and virus at 37 °C, washed with PBS and replaced by fresh medium. For all assays, CHIKV replication was measured by *nanoluc* activity at 16 h.p.i.. Schematic representation of each time-based assay as indicated by BHK-21 cells (black bars), PLA2_CB_ (grey bars), and CHIKV-*nanoluc* (blue bars), CHIKV and PLA2_CB_ inoculum (microtube), incubation at 4 °C (ice crystal) and incubation at 37 °C (thermometer). Mean values ± SD of a minimum of three independent experiments each measured in triplicate. (****) P < 0.0001. All images were generated using GraphPad Prism 8 and GIMP 2.10.20 (www.gimp.org).

To further evaluate the PLA2_CB_ effect on CHIKV entry to the host cells, virus and PLA2_CB_ were simultaneously added to BHK-21 cells for 1 h at 37 °C, cells were washed with PBS and replaced with fresh medium (Fig. 3A). PLA2_CB_ demonstrated to decrease 95.3% of CHIKV replication (p < 0.0001), indicating that this compound strongly inhibited the CHIKV-*nanoluc* entry (Fig. 3A). Combining this treatment with 1 h pre-incubation of the inoculum containing PLA2CB and CHIKV at 37 °C further increased inhibition that reached over 99% (Fig. 3B), indicating that PLA2_CB_ also possesses virucidal activity. To analyze the effect of PLA2_CB_ on CHIKV attachment, virus and compound were first incubated with the cells at 4 °C for 1 h. At this temperature, virus particles were able to attach to the cellular receptors, but not entry into the host cells. Cells were then washed with PBS, fresh medium added, and incubated at 37 °C (Fig. 3C), to allow the continuation of the entry process. Data obtained from this assay also showed strong inhibition of CHIKV attachment by reducing virus entry by 98.2% to the cells (p < 0.0001) (Fig. 3C). Post-attachment was evaluated by including an additional incubation of 30 min at 37 °C to the previous protocol (Fig. 3D), showing that the inhibition reminded strong reaching 95.2% (p < 0.0001) (Fig. 3D). Taken together this data indicates that PLA2_CB_ possesses a robust virucidal activity and the ability to block virus entry to host cells.

### PLA2_CB_ moderately affect post-entry steps of CHIKV infection

**Figure 4:**
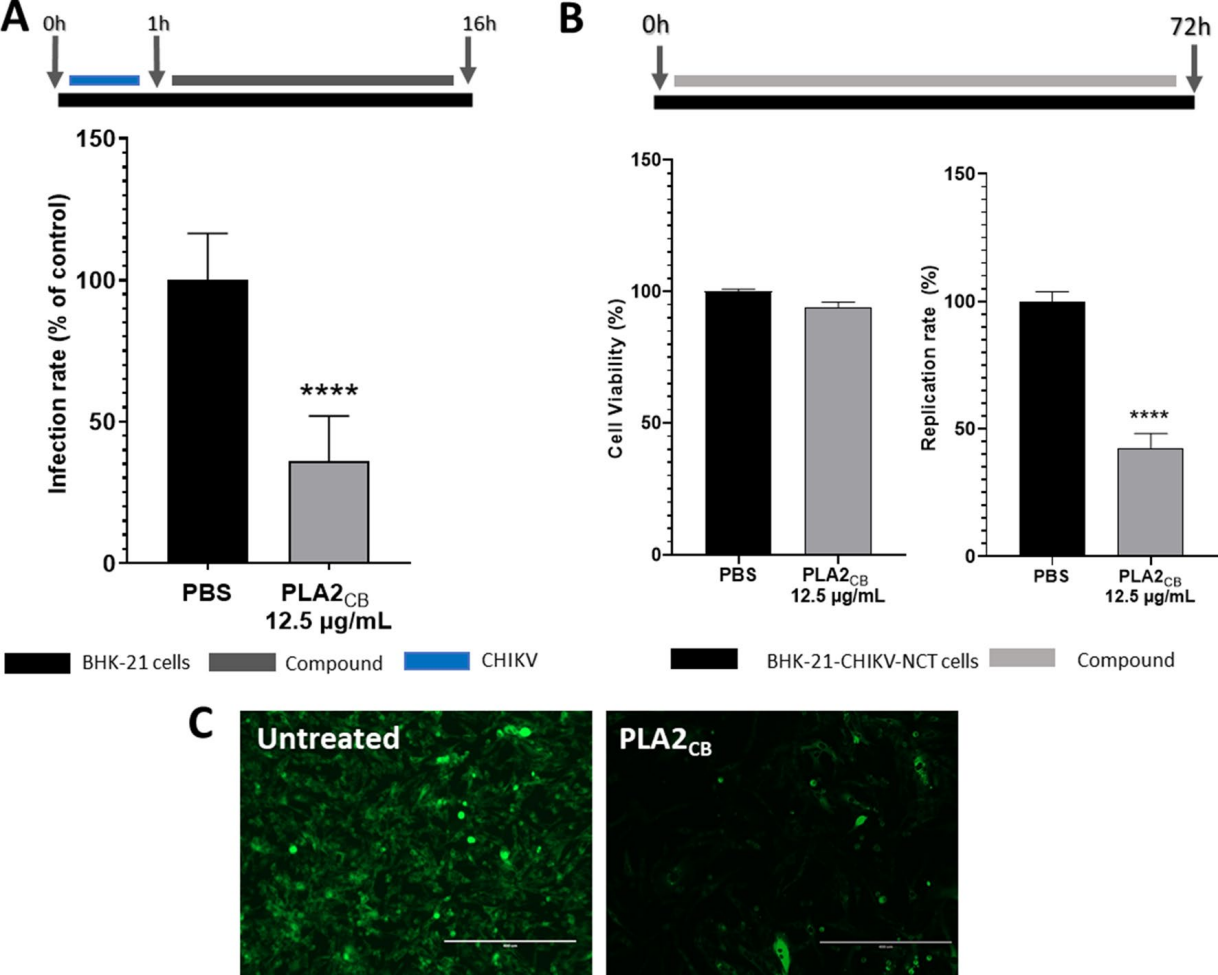
PLA2_CB_ inhibits post-entry stage of CHIKV. (**A**) BHK-21 cells were infected with CHIKV-*nanoluc* (MOI 0.1) for 1 h, washed to remove unbound virus and added of medium containing PLA2_CB_ at 12.5 μg/mL for 16 h. *Nanoluciferase* levels were measured to access CHIKV-*nanoluc* replication rates. (**B**) BHK-CHIKV-NCT cells were seed 24 h prior treatment and treated with PLA2_CB_ at 12,5 μg/mL for 72 h. *Renilla* luciferease activity and cellular viability measured. (**C**) Fluorescence of untreated control and PLA2_CB_ treatment in BHK-CHIKV-NCT, observed in fluorescence microscopy using 20 × lens (scale bar 400 µm), in GFP filter. Schematic representation of each assay as indicated by BHK-21 cells or BHK-CHIKV-NCT (black bars), PLA2_CB_ (grey bars), and CHIKV-*nanoluc* (blue bars). Mean values ± SD of a minimum of three independent experiments each measured in triplicate. (****) P < 0.0001. All images were generated using GraphPad Prism 8 and GIMP 2.10.20 (www.gimp.org).

Two assays were used to analyze effects of PLA2_CB_ on post-entry stages of CHIKV infection. Using CHIKV-*nanoluc* it was found that, if added after virus infection, compound cause relatively modest, 64% reduction of CHIKV replication (p < 0.0001) (Fig. 4A). To reveal the effect of PLA2_CB_ on virus RNA replication in the absence of production and spread of virions, BHK-CHIKV-NCT cells were used. This stable cell line contains CHIKV replicon which continuously expresses viral nonstructural proteins and two reporters: *Renilla* luciferase from nonstructural region and EGFP via activity of viral subgenomic promoter. Measurement of activities of these reporters allows the evaluation of the effect of PLA2_CB_ on replication complexes formed during the replication stage as well as on production and translation of subgenomic RNAs. Treatment of BHK-CHIKV-NCT cells with PLA2_CB_ at 12.5 µg/mL for 72 h after treatment revealed the reduction of *Renilla* luciferase expression by 58% without causing detectable cytotoxicity (Fig. 4B); this data confirms observation made using CHIKV-*nanoluc*. Furthermore, the levels in EGFP expression were also reduced as seen in Fig. 4C, indicating decrease of subgenomic RNA synthesis and/or translation. Taken together, these results suggest that PLA2_CB_ inhibits post-entry stages of infection possibly interfering with functioning of CHIKV nonstructural proteins.

### Molecular docking reveals possible interactions between PLA2_CB_ and CHIKV glycoproteins

**Figure 5:**
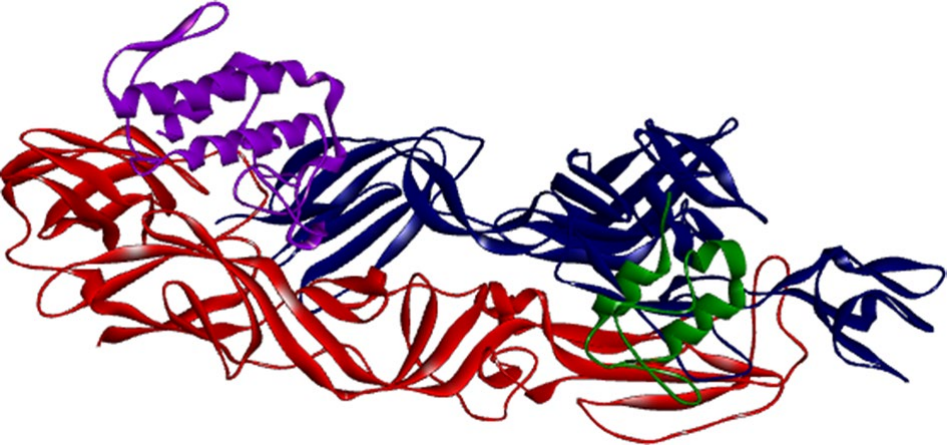
Molecular docking suggests possible interactions between PLA2CB and CHIKV glycoproteins. The post-docking 3D image was generated in the DS Visualizer program (Dassault Systèmes BIOVIA, Discovery Studio Visualizer, 2016). The CHIKV envelope glycoproteins E1 (Red), E2 (Blue), E3 (green), complexed with PLA2_CB_ (purple) are shown. All images were generated using GraphPad Prism 8 and GIMP 2.10.20 (www.gimp.org).

Results of inhibition assays clearly demonstrate that PLA2_CB_ inactivates CHIKV virions and impairs their binding to host cell suggesting interaction of compound with outer surface of virion. Therefore, a molecular docking assay was performed to investigate interactions and reveal potential binding mode between PLA2_CB_ and CHIKV glycoproteins. In a blind molecular docking, PLA2_CB_ was predicted to interact with the E1 and E2 of the glycoprotein complex, with global energy of − 0.57 kJ/mol after refining (Fig. 5).

**Figure 6:**
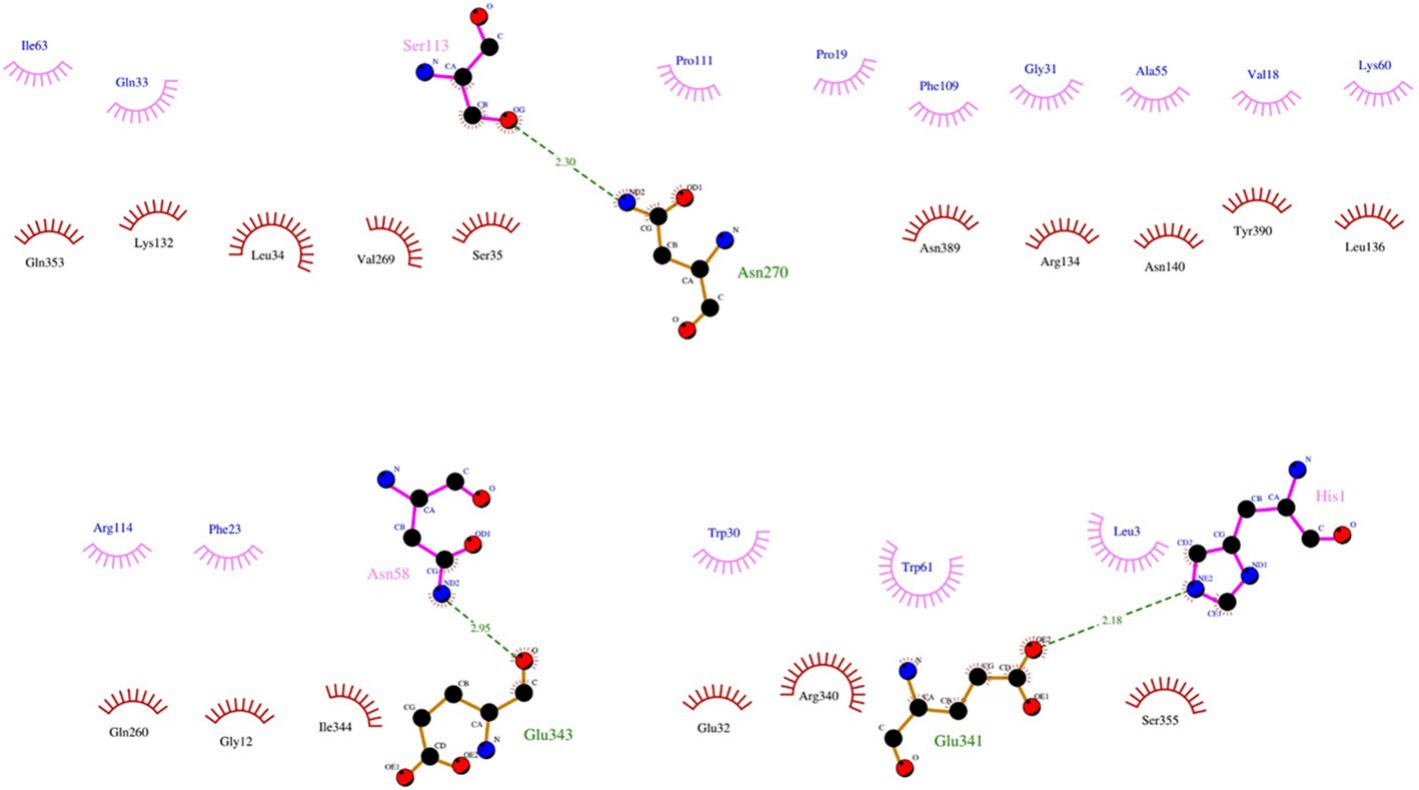
Molecular interactions between CHIKV E1 and PLA_CB_. 2D diagram of the interactions of the envelope glycoprotein E1 protein with PLA2_CB_. The hydrogen bonds (green dashes) are shown between PLA2_CB_ (purple lines) and E1 glycoprotein (orange lines). Hydrophobic interactions are also shown between PLA2_CB_ (purple bows) and E1 glycoprotein (red bows). All images were generated using GraphPad Prism 8 and GIMP 2.10.20 (www.gimp.org).

**Figure 7:**
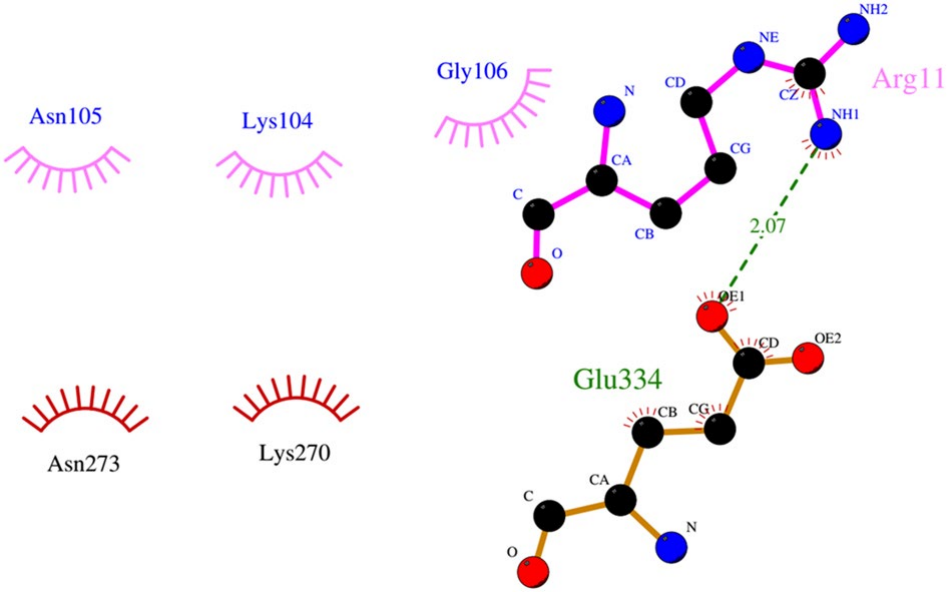
Molecular interactions between CHIKV E2 and PLA_CB_. 2D diagram of the interactions of the envelope glycoprotein E2 protein with PLA2_CB_. The hydrogen bonds (green dashes) are shown between PLA2_CB_ (purple lines) and E1 glycoprotein (orange lines). Hydrophobic interactions are also shown between PLA2_CB_ (purple bows) and E1 glycoprotein (red bows). All images were generated using GraphPad Prism 8 and GIMP 2.10.20 (www.gimp.org).

The 2D interactions between PLA2_CB_ and CHIKV glycoproteins showed that PLA2_CB_ mainly interacts with E1 glycoprotein, forming thirty hydrophobic interactions (residues Ile63, Gln33, Pro19, Phe109, Gly31, Ala55, Val18, Lys60, Arg114, Phe23, Trp30, Trp61, Leu3 in PLA2_CB_ and residues Gln353, Lys132, Leu34, Val269, Ser35, Asn389, Arg134, Asn140, Tyr390, Leu136, Gln260, Gly12, He344, Glu32, Arg340, Ser355 in E1 glycoprotein) (Fig. 6). Also, PLA2_CB_ formed 3 hydrogens bonds with E1, Ser113 and Asn270 (2.30 Å), Asn58 and Glu343 (2.95 Å) and His1 and Glu341 (2.18 Å) (Fig. 6). PLA2_CB_ may also form one hydrogen bond with E2 glycoprotein (between Arg11 and Glu 334 (2.07 Å), plus five hydrophobic interactions (Asn105, Lys104, Gly106 in PLA2_CB_ and Asn273, Lys270 in E1) (Fig. 7).

### PLA2_CB_ causes molecular changes in CHIKV glycoprotein

**Figure 8:**
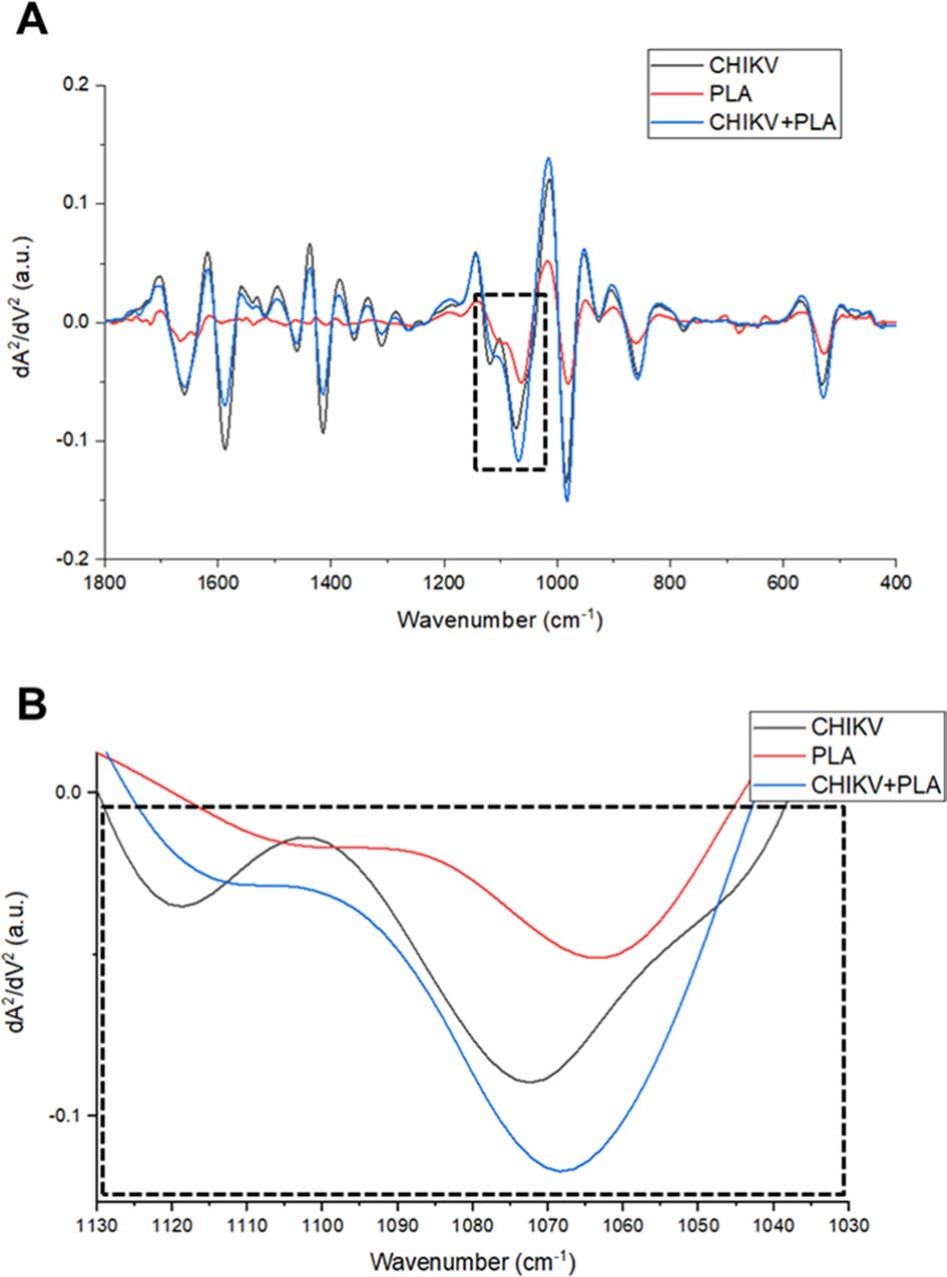
Infrared spectroscopy Spectral analysis indicates interactions between CHIKV virion and PLA_CB_. (**A**) Representative infrared average spectrum of second derivative analysis from PLA2_CB_ (red line), CHIKV virion (black line), and PLA2_CB_ plus CHIKV virion (blue line) employing an Fourrier Transform Infrared (FTIR) methodology. (**B**) Second derivative analysis, which the value heights indicate the intensity of each functional group. All images were generated using GraphPad Prism 8 and GIMP 2.10.20 (www.gimp.org).

**Figure 9:**
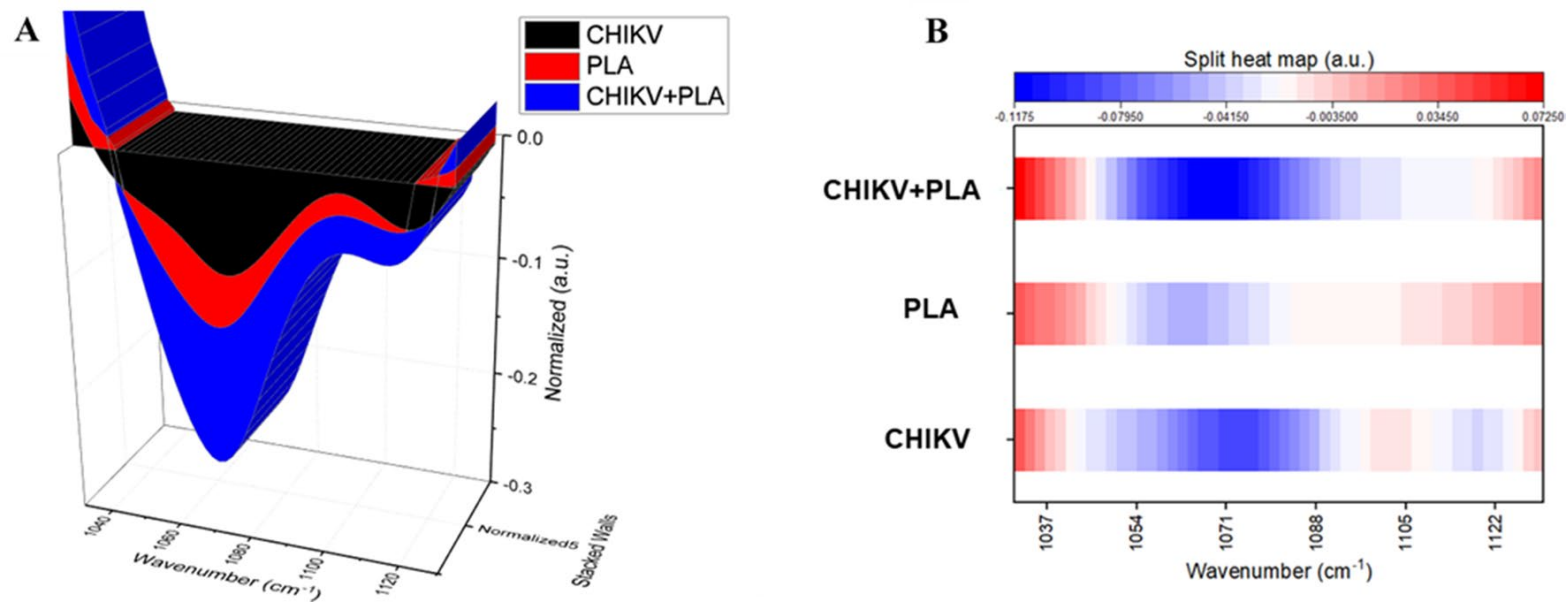
Infrared spectroscopy Spectral analysis between CHIKV virion and PLA_CB_. A representative Stacked Walls (**A**) and split heat map (**B**) of the infrared average spectrum of second derivative analysis from PLA2_CB_ (red), CHIKV virions (black), and PLA2_CB_ plus CHIKV virions (blue). All images were generated using GraphPad Prism 8 and GIMP 2.10.20 (www.gimp.org).

To further investigate the interactions between PLA2_CB_ and CHIKV particles, infrared spectroscopy spectral analysis and vibrational analysis among the virus and PLA2_CB_ was performed. Representative means of the infrared spectrum of CHIKV, PLA2_CB,_ and CHIKV plus PLA2_CB_, which is the bio fingerprint region representing proteins, lipids, nucleic acids, and glycoproteins are shown in Fig. 8A. A representative infrared average spectrum of second derivative analysis from CHIKV virions, PLA2_CB,_ and CHIKV virions plus PLA2_CB_ is displayed in Fig. 8A. In the second derivative analysis, the value heights indicate parallel changes in the intensity of each functional group. The binding interaction between CHIKV virions and PLA2_CB_ was mainly revealed by the increase in the vibrational mode at 1068 cm^−1^, which indicates detection of additional stretching of C-O ribose present in glycoprotein derived from the association CHIKV virions and PLA2_CB_^38–40^ (Fig. 8B). Furthermore, the Stacked Walls (Fig. 9A) and split heat map (Fig. 9B) reinforces the additional expression of vibrational mode at 1068 cm^−1^ under CHIKV virions plus PLA2_CB_ association.

## Discussion

Natural PLA2_CB_ has shown to have broad spectrum antiviral activity^32–34^. Here, we assessed the antiviral activity of the PLA2_CB_ against CHIKV, as well as sought comprehension on its mechanism of action. Our results demonstrated that PLA2_CB_ strongly inhibited CHIKV infection, corroborating with Russo and colleagues work, which demonstrated that rPLA2_CB_ impaired CHIKV infection^35^. Additionally, the results demonstrated that the pre-treatment of naïve cells with PLA2_CB_ protected host cells against CHIKV infection. In accordance with our study, Chen and coworkers have reported that phospholipase A2 isolated from the venom of the honeybee *Apis mellifera* was able to protect cells against Human immunodeficiency virus (HIV) and dengue virus (DENV) infections^41^. Fenard and colleagues also demonstrated that cells can be protected against HIV infection by different phospholipases A2 isolated from several mammalian species^42^. The PLA2s from snake venoms are classified in the group II of a secreted family phospholipases and show homology to the mammalian inflammatory PLA_2_, which play different roles in the organism including in an immune response to infectious diseases^43–46^. Therefore, our data might also suggest that PLA2_CB_ plays a role in host cell metabolism and as a result protects cells against viral infection, by the possible mimicking effect of phospholipases found in host cells.

Our findings that PLA2_CB_ has strong virucidal effect and interferes with viral entry to the host cells are consistent with previous findings made for two flaviviruses, DENV and yellow-fever (YFV)^34^. The authors demonstrated that incubation of DENV or YFV virions with PLA2_CB_ results in inhibition of early steps of viral infection probably by disrupting virion envelope membrane and/or blocking virus adsorption^33,34^. Additionally, Russo and coworkers described that incubation of rPLA2_CB_ with CHIKV prior to the infection of cells significantly impaired CHIKV infectivity^35^. Therefore, our results are in agreement with previous data that suggested that the predominant activity of PLA2_CB_ is due to its virucidal effect, probably by acting on the virus particle. Several PLA2s isolated from snake venom have been described to possess antiviral activity against DENV, YFV, Herpes simplex types 1 and 2 and Influenza A (H3N2) by interacting with lipid membrane found in a pocket between glycoproteins and/or through attachment to the glycoproteins in the viral envelope surface^33,34,41,47^. Based on this data, we performed a blind molecular docking using PLA2_CB_ and the CHIKV glycoproteins complex (E1, E2, and E3) to assess the possible interaction among then. The results demonstrated that PLA2_CB_ most likely bonded to both E1 and E2 with E1 being the main target. These results are also consistent with the virucidal effect described here and corroborate previously published data^35^. The glycoproteins E1 and E2 are essential during the early stages of CHIKV infection. The CHIKV glycoprotein E2 is responsible for binding to cells receptors such as MXRA^48^; the binding occurs in the “canyon” between protomers of CHIKV spike complex. In addition, E1 is a viral fusion protein that ensures the envelope fusion with host cells membranes^49,50^. Thus, molecules that can interact with E1 and/or E2 attachment sites can prevent virus entry^51^. We propose that PLA2_CB_ might act be binding to E1 and/or E2 glycoproteins and preventing virion entry by impairing attachment to the cells and/or membrane fusion. PLA2_CB_/glycoproteins interactions, characterized using an infrared spectrum assay, did indeed support the hypothesis of interaction between CHIKV virions and PLA2_CB_. On the other hand, it is important to emphasize that alphavirus virions exist in two state with different characteristics, a normal infectious state and pre-fusion state when E1 is activated prior to attachment^52–54^. In this context, the employment of cryogenic electron microscopy (cryo-EM) or atomic force microscopy presents as potential approaches to better characterize this interaction.

Moreover, PLA2_CB_ also demonstrated a modest yet significant ability of inhibit post-entry steps of CHIKV infection. Using CHIKV subgenomic replicon it was found that both RNA replication and subgenomic RNA synthesis were inhibited. Shimizu and coworkers have previously reported that PLA2_CB_ also affected the post-entry steps of HCV infection^32^, corroborating with our findings. The mode how PLA2_CB_ affects RNA replication step remains currently unknown. However, it can be speculated that this may be associated with its effect on cell membranes that are required for formation of RNA replicase complexes. The exact mechanism of this as well as similarities and differences between anti-HCV and anti-CHIKV effects represent topics for additional studies.

In summary, our study evidenced that PLA2_CB_ isolated from *Crotalus durissus terrificus* inhibited multiple steps of CHIKV infection. The enzyme was able to protect the target cells against CHIKV infection, impaired virus entry to the host cells, mainly by virucidal activity, and also disturbed post-entry steps of the CHIKV infection. Therefore, this data might be useful for further development of new antiviral approaches against CHIKV and provide the potential for treatment of Chikungunya fever.

## Methods

### Compound

The crude venom of *Crotalus durissus terrificus* was obtained from the “Animal Toxin Extraction Center” (CETA), duly registered and approved by the Ministry of the Environment under de process number 3002678. The venom was collected from 28 specimens from the Morungaba—SP collection under the Brazilian Institute for the Environment and Renewing Natural Resources (IBAMA) authorization: 1/35/1998/000846–1, and extraction was performed by Jairo Marques do Vale (CETA). All experiments were performed in accordance with relevant named guidelines and regulations available in the federal universities, IBAMA and the Ministry of Environment. The isolation and purification of phospholipase PLA2_CB_ (Fig. 1A) from the venom of *Crotalus durissus terrificus* snakes were carried out at the Toxinology Laboratory of the School of Pharmaceutical Sciences of Ribeirão Preto, University of São Paulo, as previously described^28,34^. The lyophilized protein was dissolved in PBS (phosphate buffer saline), filtered, and stored at -80ºC. Dilutions of the stock solution containing the protein were made immediately prior to the experiments. For all the performed assays, PBS was used as the untreated control. All authors complied with the ARRIVE guidelines.

### Cell culture

BHK-21 cells (fibroblasts derived from Syrian golden hamster kidney; ATCC CCL-10), purchased from The Global Bioresource Center (ATCC), were maintained in Dulbecco’s modified Eagle’s medium (DMEM, SIGMA-ALDRICH) supplemented with 100U/mL of penicillin (HYCLONE LABORATORIES), 100 mg/mL of streptomycin (HYCLONE LABORATORIES), 1% dilution of stock of non-essential amino acids (Hyclone Laboratories) and 1% of fetal bovine serum (FBS, HYCLONEN LABORATOIRES) in a humidified 5% CO_2_ incubator at 37 °C. Subgenomic replicon (SGR) harboring cell lines (BHK-CHIKV-NCT)^37^ were maintained under the same conditions of BHK-21 cells (ATCC CCL-10), except for the addition of G418 (SIGMA-ALDRICH) at 5 mg/mL.

### Rescue of CHIKV-*nanoluc* reporter virus

The CHIKV expressing *nanoluciferase* reporter (CHIKV*-nanoluc*) (Fig. 1B) used for the antiviral assays is based on the CHIKV isolate LR2006OPY1 (East/Central/South African genotype). The infectious cDNA of CHIKV-*nanoluc* was placed under control of the CMV promoter^36^. To produce CHIKV-*nanoluc* virions, 2.3 × 10^7^ BHK-21 cells seeded in a T175 cm^2^ flask were transfected with 1.5 μg of CHIKV-CMV-*nanoluc* plasmid using Lipofectamine 2000 and Opti-Mem medium. 48 h post-transfection (h.p.t.) the supernatant was collected and stored at − 80 °C. To determine viral titers, 1 × 10^5^ BHK-21 cells were seeded in each of wells of 24 wells plate; 24 h later the cells were infected with tenfold serially dilutions of CHIKV*-nanoluc.* Cells were incubated with virus for 1 h 37 °C; after this, the inoculums were removed, cells were washed with PBS to remove the unbound virus, and fresh medium supplemented with 1% dilution of stock of penicillin and streptomycin, 2% FBS and 1% carboxymethyl cellulose (CMC) was added. Infected cells were incubated for 2 days in a humidified 5% CO_2_ incubator at 37 °C, followed by fixation with 4% formaldehyde and staining with 0.5% violet crystal. The viral foci were counted to determine viral titer which was presented in plaque forming units per milliliter (PFU/mL).

### Cell viability assay

Cell viability was measured by MTT [3-(4,5-dimethylthiazol-2-yl)-2,5-diphenyl tetrazolium bromide] (SIGMA-ALDRICH) assay as previously described^55^. BHK-21 cells were plated to 48 well plates at a density of 5 × 10^4^ cells per well and incubated overnight at 37 °C. Medium containing two-fold serial dilutions of PLA2_CB_ (from 0.195 to 200 µg/mL) was added and cells were incubated for 16 h. After this, the medium was replaced with the MTT solution at 1 mg/mL, cells were incubated for 30 min, after which MTT solution was and replaced with 300 μL of DMSO (dimethyl sulfoxide) to solubilize the formazan crystals. The absorbance was measured at 490 nm on the Glomax microplate reader (PROMEGA). Cell viability was calculated according to the equation (T/C) × 100%, where T and C represent the mean optical density of the treated and untreated control groups, respectively. The cytotoxic concentration of 50% (CC_50_) was calculated using GraphPad Prism 8.

### Determination of the effective concentration 50% (EC_50_)

To assess the antiviral activity of PLA2_CB_, BHK-21 cells were seeded at a density of 5 × 10^4^ cells per well into 48 well plates for 24 h and infected with CHIKV-*nanoluc* at a multiplicity of infection (MOI) of 0.1 PFU/cell as described by Oliveira and coworkers^55^. The PLA2_CB_ at concentrations ranging 0.195–200 µg/mL was added to growth media. Samples were harvested using Renilla-luciferase lysis buffer (PROMEGA) at 16 h post-infection (h.p.i.) and virus replication levels were quantified by measuring *nanoluciferase* activity using the Renilla luciferase Assay System (PROMEGA). The effective concentration of 50% inhibition (EC_50_) was calculated using GraphPad Prism 8 software. The values of CC_50_ and EC_50_ were used to calculate the selectivity index (SI = CC_50_/EC_50_).

### Time-of-addition assays

BHK-21 cells at the density of 5 × 10^4^ cells per well were seeded in 48 well plates 24 h before infection and treatment. All infections were performed at MOI of 0.1 and efficiency of virus replication was assessed by measurement of *nanoluciferase* activity at 16 h.p.i.

In pretreatment assay, cells were treated for 1 h with the compound prior to the CHIKV infection, extensively washed with PBS and added of CHIKV*-nanoluc* for 1 h. Then, cells were washed with PBS and incubated to remove unbound virus and added of fresh medium for 16 h (Fig. 2A). Alternatively, cells were treated for 1 h with the compound, washed with PBS and infected with CHIKV-*nanoluc* at the presence (Fig. 2B) of PLA2_CB_ for 16 h.

In entry inhibition assay, cells were infected using media containing the compound- and virus for 1 h, washed with PBS and incubated with fresh medium for 16 h (Fig. 3A). The virucidal activity was assessed using the same setting except inoculum containing compound and virus was incubated for 1 h before it was added to the cells (Fig. 3B). The impact of compound on attachment step was analyzed using the same setting as in entry inhibition assay except cells were incubated with virus and compound at 4 °C (Fig. 3C). A variant of this assay where the incubation at 4 °C was followed by incubation for 30 min at 37 °C was used to analyze the effect of compound on post-attachment steps of infection (Fig. 3D).

In post-entry assay, cells were infected with CHIKV for 1 h, washed extensively with PBS, and the incubated in compound-containing medium for 16 h (Fig. 4A).

### RNA replication assay using BHK-CHIKV-NCT cells

BHK-CHIKV-NCT cells that express CHIKV nonstructural proteins, a selection marker (puromycin acetyltransferase, Pac) and *Renilla* luciferase and EGFP reporters^37^, were used to assess the activity of PLA2_CB_ on CHIKV RNA replication. Cells were seed at a density of 7 × 10^3^ cells per well of a 96 well plate. After 24 h, cells were treated with the PLA2_CB_ at 12.5 µg/mL for 72 h (Fig. 4B). The impact of compound on CHIKV RNA replication was estimated by quantification of *Renilla* luciferase expression. In addition, EGFP fluorescence was monitored by placing plates directly using an EVOS (THERMO-FISCHER) fluorescence microscope and using 20 × lens and GFP filter.

### Molecular docking analysis

The interaction between PLA2_CB_ (PDB: 3R0L) and the envelope glycoproteins of the CHIKV (PDB: 3N42) was analyzed using blind docking performed in the PatchDock server^56^, using the parameters predefined by the program and refined by the FireDock algorithm^57^. The best docking positions were evaluated by the geometric complementarity score defined by PatchDock, with results refined and ranked by the global energy after refinement. The post-docking 3D image was generated in the DS Visualizer program, Dassault Systèmes BIOVIA, Discovery Studio Visualizer, version 17, San Diego: Dassault Systèmes, 2016, and a 2D diagram of the interactions interface between the molecules was generated with the aid of the LigPlot + program^58^.

### Infrared spectroscopy spectral data analysis

An Fourrier Transform Infrared (FTIR) spectrophotometer Vertex 70 (BRUKER OPTICS, REINSTETTEN, Germany) connected to a micro-attenuated total reflectance (ATR) platform was used to record sample signature at 1800 cm^−1^ to 400 cm^−1^ regions as described by Oliveira and coworkers^55^. The ATR unit is composed of a diamond disc as an internal-reflection element. The sample dehydrated pellicle penetration depth ranges between 0.1 and 2 μm and depends on the wavelength, incidence angle of the beam, and the refractive index of ATR-crystal material. The infrared beam is reflected at the interface toward sample in the ATR-crystal. All samples (2µL) were dried using airflow on ATR-crystal for 3 min before sample spectra recorded in triplicate. The air spectrum was used as a background in all ATR-FTIR analysis. Sample spectra and background were taken with 4 cm^−1^ of resolution and 32 scans were performed for analysis. The spectra were normalized employing the vector method and adjusted to rubber band baseline correction. The original data were plotted in the Origin Pro 9.0 (ORIGINLAB, Northampton, MA, USA) software to create the second derivative analysis. The second derivative was obtained by applying the Savitzky-Golay algorithm with polynomial order 5 and 20 points of the window. The value heights indicated the intensity of the functional group evaluated.

### Statistical analysis

Individual experiments were performed in triplicate and all assays were performed a minimum of three times to confirm the reproducibility of the results. GraphPad Prism 8 software was used to assess statistical differences of means of readings using Student’s unpaired t-test or Mann–Whitney tests. P values < 0.01 were considered to be statistically significant.
